# Characterizing the boundary lateral to the shear direction of deformation twins in magnesium

**DOI:** 10.1038/ncomms11577

**Published:** 2016-06-01

**Authors:** Y. Liu, N. Li, S. Shao, M. Gong, J. Wang, R. J. McCabe, Y. Jiang, C. N. Tomé

**Affiliations:** 1 Materials Science and Technology Division, Los Alamos National Laboratory, Los Alamos, New Mexico 87545, USA; 2 Materials Physics and Applications Division, Los Alamos National Laboratory, Los Alamos, New Mexico 87545, USA; 3 Department of Mechanical and Materials Engineering, University of Nebraska–Lincoln, Lincoln, Nebraska 68583, USA; 4 Department of Mechanical Engineering, University of Nevada, Reno, Reno, Nevada 89557, USA

## Abstract

The three-dimensional nature of twins, especially the atomic structures and motion mechanisms of the boundary lateral to the shear direction of the twin, has never been characterized at the atomic level, because such boundary is, in principle, crystallographically unobservable. We thus refer to it here as the dark side of the twin. Here, using high-resolution transmission electron microscopy and atomistic simulations, we characterize the dark side of 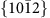 deformation twins in magnesium. It is found that the dark side is serrated and comprised of 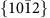 coherent twin boundaries and semi-coherent twist prismatic–prismatic 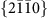 boundaries that control twin growth. The conclusions of this work apply to the same twin mode in other hexagonal close-packed materials, and the conceptual ideas discussed here should hold for all twin modes in crystalline materials.

## Abstract (web summary)

Twin propagation involves three-dimensional normal, forward and lateral motion of twin interfaces with respect to the twinning shear direction. Here, the authors combine electron microscopy and atomistic simulations to study the until now unknown lateral structure of tensile deformation twins in magnesium.

## Graphical abstract

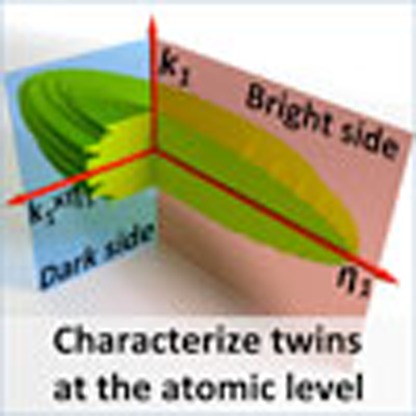

Due to the scarcity of ‘easy slip' systems, twinning is a major plastic deformation mode in hexagonal close-packed (hcp) metals at room temperature^1^,^2^, and influences the ductility and formability of hcp metals^3^,^4^,^5^,^6^. As a consequence, basic knowledge of the motion mechanisms of twin boundaries (TBs) should help us to develop better alloys, processing routes and material models for the purpose of designing materials with the desired properties and microstructures^7^,^8^,^9^,^10^,^11^,^12^. The commonly activated 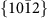 twin in hcp metals is a compound twin with the characteristics of both, type I and type II twins: a rational crystallographic twin plane *K*_1_, and a rational shear direction *η*_1_, respectively. The compound twin transformation consists of either a rotation of 180° around the normal to the twin plane *K*_1_ or, equivalently, around the *η*_1_ direction^1^. As a consequence of the transformation, one set of prismatic planes in the twin domain remains parallel to one set of prismatic planes in the matrix, but the orthogonal basal planes at each side of the boundary are twisted about the prismatic normal by 93.78° with respect to each other in magnesium (Mg). The propagation of 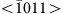 twinning dislocations (TDs) in alternating 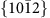 planes, combined with an atomic shuffle, has the effect of growing twins perpendicular to the 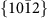 plane and along the 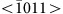 twinning direction^13^,^14^,^15^,^16^,^17^,^18^. Surprisingly, there is no reference in the literature about the lateral growth of the twin, although such mobility can be expected to condition greatly the overall propagation of the twin and its ability to accommodate shear in a meaningful volume of the grain. A possible reason for such neglect may be that microscopy characterization of the dark side (DS) is not trivial.

In the following, we identify and characterize the DS of 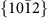 twins using high-resolution transmission electron microscopy (HRTEM) and atomistic simulations, and find that the DS of twins is formed by coherent TBs (CTBs) and semi-coherent twist prismatic–prismatic (T-PP2) boundaries.

## Results

### Bright side of 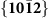 twins

**Figure 1:**
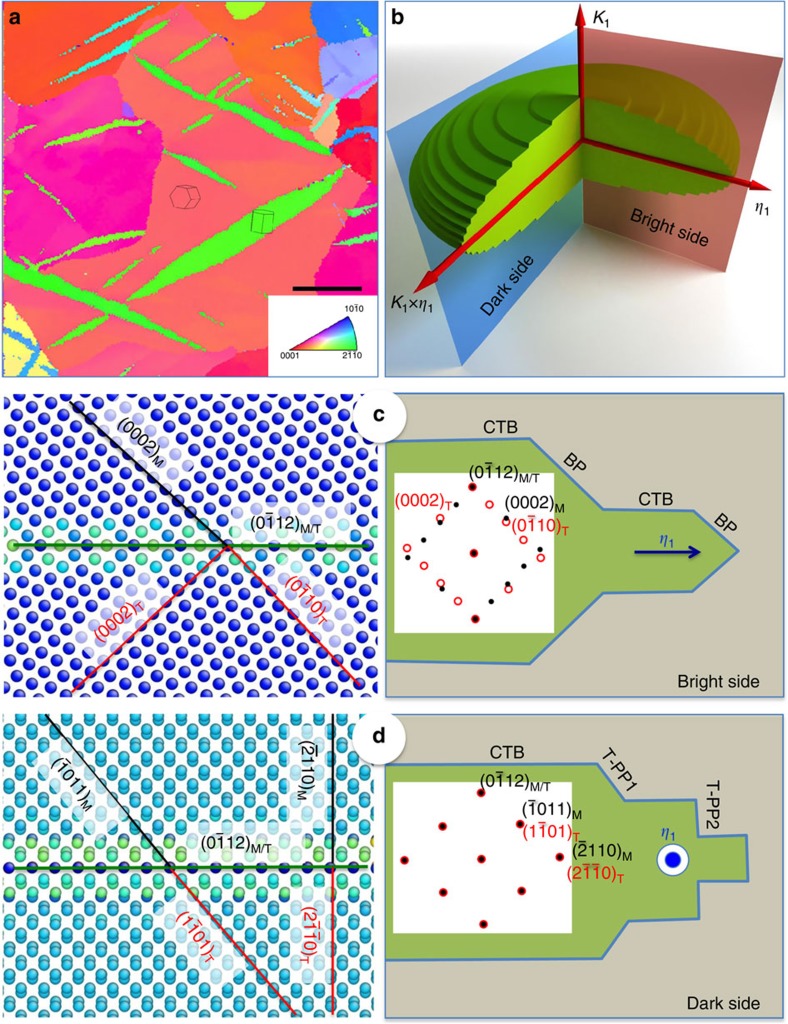
Three-dimensional twin boundaries of 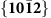 twins. (**a**) Electron-backscatter diffraction analysis of deformed polycrystalline magnesium shows twins of elliptical shape with the long axis along the twinning direction. Scale bar, 40 μm. (**b**) Speculated ellipsoidal morphology of a three-dimensional twin. The bright side refers to the observation along a direction perpendicular to both the twinning shear direction (*η*_1_) and the normal to the twin plane (*K_1_*). The dark side refers to the observation along twinning shear direction (*η*_1_). (**c**) The bright side of a 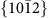 deformation twin domain shows two types of boundaries: coherent twin boundaries (CTBs) (atomic structure on left side) and prismatic||basal boundaries (PB/BP). A schematic of these twin boundaries (CTBs and PB/BPs) and corresponding electron diffraction pattern is on right side. (Details in Supplementary Fig. 2). (**d**) Speculated characteristics of the dark side of a 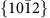 deformation twin domain: CTBs (atomic structure on left side) twist pyramidal–pyramidal (T-PP1) and twist prismatic–prismatic (T-PP2) boundaries with high atomic areal density. A schematic of these twin boundaries and corresponding electron diffraction pattern is on right side. In principle both, twin and matrix domains, have identical lattice and diffraction pattern, which makes characterization of this side view of twins difficult at the atomic level.

Experimental characterizations using electron backscatter diffraction (EBSD) or TEM in two-dimensional sections show an approximately elliptical twin shape, with the long axis along the twinning direction (Fig. 1a). Thus, the three-dimensional (3D) shape is approximately ellipsoidal (Fig. 1b), although 3D EBSD grain reconstruction suggests that twin variants with small Schmid factor may have more tortuous shapes^19^ (Supplementary Fig. 1). The growth of the compound twins mostly involves the glide of TDs, although a significant contribution to growth by atomic shuffle has been reported for micron-sized single crystals of cobalt and Mg^18^,^20^. When TBs are observed along a direction perpendicular to the twinning (shear) direction *η*_1_ and the normal to the twin plane *K*_1_ two typical TBs are characterized, CTBs parallel to the twinning plane and prismatic||basal boundaries (PB/BPs) associated with pile-up and rearrangement of TDs (Fig. 1c). The former is also referred to as a symmetric tilt boundary, while the latter is referred to as an asymmetric tilt boundary. These TBs have been characterized using HRTEM and atomistic simulations in hcp metals (Supplementary Fig. 2)^20^,^21^,^22^,^23^,^24^. This view is here referred to as bright side (BS) of a twin domain. HRTEM characterization of the BS, in particular, has provided insight into nucleation and growth mechanisms of 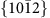 twins in hcp metals and the influence of TBs on mechanical responses. Under some circumstances, the migration of PB/BPs can dominate twin growth resulting in significant deviations of the TB away from the twinning plane^18^,^20^,^25^. More surprisingly, PB/BPs have been shown to be responsible for twin nucleation via a pure-shuffle mechanism due to their lower interface energy than CTBs^26^. The latter is a deviation from the classic dislocation-based nucleation mechanisms. During cyclic loading, twin–twin junctions form that contain tilt prismatic–prismatic and basal–basal boundaries that suppress or delay detwinning and lead to strain hardening^27^. In addition, TBs have distinctive structures that may lead to different solubility of solute atoms. Using first principle density function theory calculation, Kumar *et al*.^9^ studied the solubility of different solute atoms in CTBs and PB/BPs as a function of local stresses. Nie *et al*.^8^ have experimentally characterized periodic segregation of solute atoms along CTBs and PBs, and demonstrated the pinning effect of solute atoms on TBs during mechanical loading, suppressing twin growth while strengthening materials.

### Dark side of 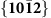 twins

When the twin domain is observed along the twinning direction *η*_1_, the twin and the matrix's selected area diffraction (SAD) patterns will appear identical (Fig. 1d). In addition, the projection on the observation plane of atom columns in both domains is also identical (Fig. 1d). This makes characterization of this view of twins difficult at the atomic level, although the morphology of such special TBs in type II twins can be observed based on local strain contrast^28^,^29^. The DS of twins might be revealed by TEM at the atomic level if twist TBs in the DS view relax to form semi-coherent interfaces associated with the formation of misfit dislocations. These misfit dislocations will cause local elastic distortion and, as a consequence, the twin and matrix across the coherent boundary will deviate locally from the perfect twin orientation, although they retain the twin orientation in the far field^30^. For example, disclination dipoles form in the corners of the PB/BP steps that accommodate the rotation of 3.78° (in Mg twinning corresponds to 86.22° rotation while PB corresponds to a 90° rotation)^13^,^31^,^32^.

### Atomistic simulations of the dark side of 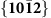 twins

**Figure 2:**
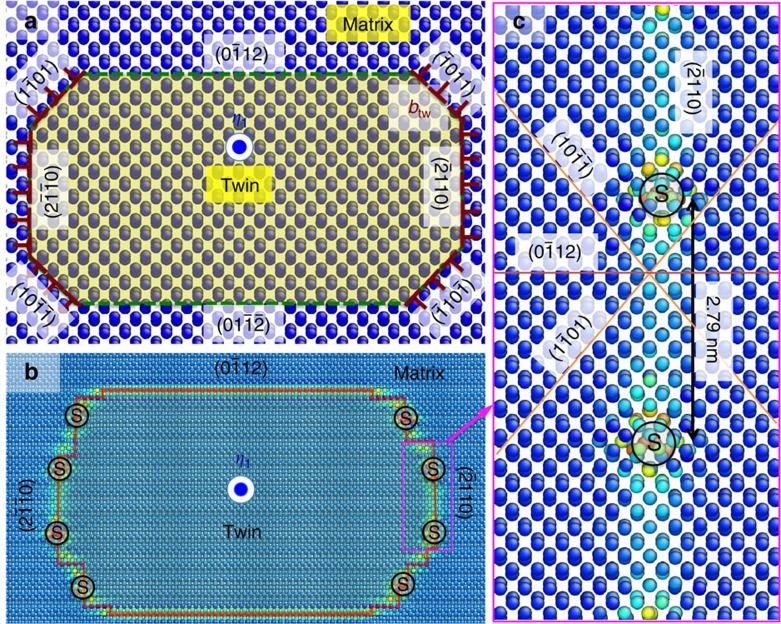
Atomistic simulation of atomic structures of the dark side. (**a**) The atomic structure of the dark side along the twinning direction (*η*_1_). Three atomic planes, 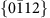 (CTB), 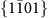 (T-PP1) and 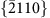 (T-PP2), with high atomic areal density are chosen to be potential low-energy interfaces between the matrix and the twin. (Details in Supplementary Fig. 3). (**b**) Atomic structure of a relaxed twin nucleus, showing 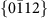 CTBs, 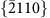 semi-coherent boundaries and discrete misfit dislocations (denoted by the black symbol ‘S') (Details in Supplementary Fig. 4 and Supplementary Note 1). (**c**) Atomic structure of T-PP2 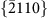 semi-coherent boundary containing coherent interface and discrete misfit dislocations. (Details on formation of T-PP2 are in Supplementary Fig. 6 and Supplementary Note 3).

Figure 2a shows a potential configuration of crystallographic interfaces corresponding to the twin orientation in the DS: CTB, twist pyramidal–pyramidal boundary (T-PP1, 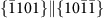) and T-PP2 boundary, (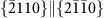). Atomistic simulations reveal the energies and atomic structures of the three interfaces: CTB has the lowest formation energy of 125 mJ m^−2^, T-PP2 has a formation energy of 212 mJ m^−2^, which is lower than most tilt grain boundaries (GBs), and T-PP1 has a formation energy of 318 mJ m^−2^, which is higher than most tilt GBs (Supplementary Fig. 3). Figure 2b shows the relaxed atomic configuration of the DS predicted by molecular dynamics (MD) simulation: observe that the twin is surrounded by CTBs and T-PP2 steps/facets (details in Supplementary Fig. 4 and Supplementary Note 1). This is consistent with the thermodynamic principle that the interface with lower energy is favoured. Figure 2c shows the atomic structures of the T-PP2 interface (details of T-PP1 and T-PP2 in Supplementary Fig. 5 and Supplementary Note 2), indicating formation of a semi-coherent interface and misfit dislocations. The formation mechanism of the semi-coherent T-PP2 interface is addressed in Supplementary Fig. 6 and Supplementary Note 3 (refs 33, 34). The Frank–Bilby formula predicts an average spacing between misfit dislocations of about 2.8 nm, which is the same as the MD result. Thus, TBs in the DS can be represented by CTB+T-PP2, where the additional twist rotation 3.78° associated with a perfect twin orientation is accommodated by misfit dislocations. This suggests that SAD patterns could show additional spots associated with the coherent interface^35^, and so provide a signature of the DS.

### Characterization of the dark side of 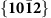 twins

**Figure 3:**
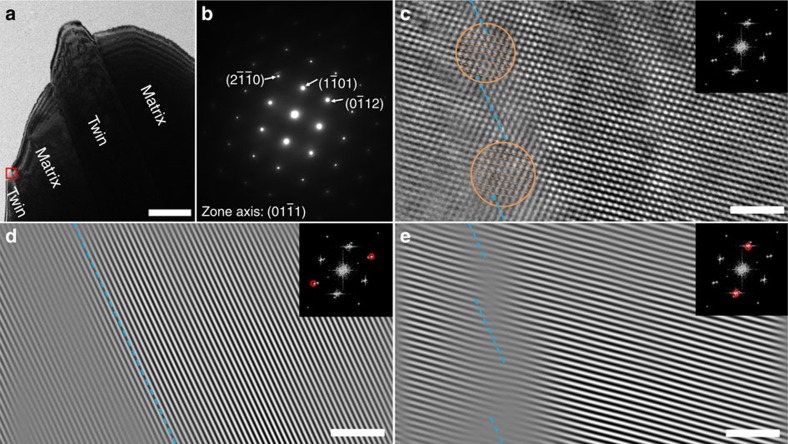
Characterization of atomic structures of the dark side. (**a**) Bright-field transmission electron microscopy image of magnesium showing two twin domains that are slightly off-zone compared with the perfectly on-zone matrix domains. Scale bar, 2 μm. (**b**) Dark side select area diffraction pattern from both the matrix and twinned regions. The twinned domains are slightly misoriented relative to the perfect twin misorientation relationship, enabling identification of twin boundaries when observed along the dark side. (**c**) High-resolution TEM and corresponding fast-Fourier transform (FFT) patterns of the red rectangle marked in **a** with boundary serrations composed of CTBs and misfit dislocations. Scale bar, 2 nm. (Details in Supplementary Fig. 9). The determination of the boundary serrations is based on the inverse FFT (IFFT) analysis of the misorientation-induced extra diffraction information for 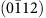 and 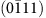 planes: (**d**) the IFFT image from 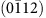 diffraction shows the well-aligned 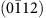 planes in the matrix domain on the right and slightly off-aligned 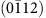 planes in twin domain on the left. Scale bar, 2 nm. The blue dash line on a 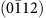 plane roughly indicates the possible boundary location; (**e**) the IFFT image from 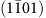 diffraction shows the discontinuity at the previously marked boundary location, which can be differentiated into several segments of CTBs. Scale bar, 2 nm. Local atomic displacements due to misfit dislocations are identified in **c** (marked with orange circle and dash-lines) and suggest the serration of the twin boundaries observing along the dark side.

We examined the DS of a 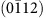 twin in Mg along the *η*_1_ direction, 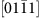 (Fig. 3a). In the far field, the DS SAD pattern sampled from both twin and parent regions appears to show a single diffraction pattern as seen in Fig. 3b. However, due to a deviation from the perfect twin relation, when the parent is perfectly aligned with the zone axis the twin domain is tilted slightly off of the zone axis. Locally, the deviation from the perfect twin relation observed by HRTEM and fast-Fourier transformations (FFTs) at the interface results in extra spots shifted slightly relative to the parent diffraction pattern. The presence of these additional spots enables us to locate the DS boundary region in the bright-field cross-sectional TEM micrograph in two steps. In the first step, we locate the TB regions, which can be clearly observed at low resolution by diffraction contrast (Fig. 3a). This is also confirmed by dark-field TEM images and local SAD patterns on both sides of the boundary (Supplementary Fig. 7; Supplementary Note 4). In step two, we characterize a small domain within the roughly identified boundary region (indicated by the red square in Fig. 3a), using HRTEM (details in Supplementary Fig. 8 and Supplementary Note 5). Using the additional spots coming from 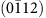 and 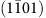, we pinpoint the boundary location (Fig. 3c–e), by identifying changes in the FFT patterns due to the twin and TB. Figure 3c shows the original HRTEM image and the corresponding FFT patterns of the marked region. The boundary is serrated and composed of CTBs (blue dash lines) and misfit dislocation cores (orange circles). Compared with the right side of the boundary, the additional spots on 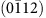 and 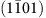 in the left side of the boundary help locate the twin-associated boundaries via the inverse FFT (IFFT) analysis in Fig. 3d–e. The IFFT–HRTEM image is filtered using the standard and deviated 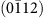 and 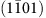 diffraction spots, (details in Supplementary Fig. 9 and Supplementary Note 6). In Fig. 3d, an IFFT image that is filtered using the standard 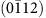 plane shows the well-aligned 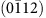 planes in the matrix domain on the right and slightly misaligned 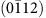 planes in twin domain on the left, to identify the possible boundary location roughly marked as a blue dash line on a 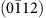 plane. In Fig. 3e, an IFFT image that is filtered using the standard 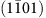 diffraction spot reveals the discontinuity at the previously marked boundary location, which is marked via several blue dash lines. The local atomic displacement can be identified as shown in orange circle in Fig. 3c and is likely related to the misfit dislocations shown in Fig. 2. It is likely that this serrated DS boundary is composed of CTBs and semi-coherent T-PP2 boundaries due to its low-energy boundary nature and less atomic displacement between misfit dislocations.

## Discussion

By using HRTEM and MD simulation, we have identified and characterized the DS of 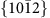 twins, and found that the DS of twins is formed by CTBs and semi-coherent T-PP2 boundaries. This finding can now be combined with our previous knowledge of the BS configuration for advancing our understanding of how 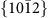 deformation twins propagate. CTBs migrate via the glide of TDs on coherent twin planes together with the associated atomic shuffles^17^; PB/BPs interfaces propagate by the glide or conservative climb of boundary dislocations with the associated atomic shuffles^13^,^22^. The findings in this work suggest that propagation of the DS of the twin involves the migration of semi-coherent T-PP2 interfaces via atomic shuffle, combined with the lateral glide of screw misfit dislocations (Supplementary Figs 4 and 5). We expect that twin propagation of the DS will be slower than the BS, and strongly dependent on temperature and strain rate due to the pinning effect of misfit dislocations on the T-PP2 interface and the shuffle aided migration of coherent regions of the T-PP2 interface. This would result in an irregular shape (as demonstrated by MD simulations in Supplementary Movie 1 and Supplementary Fig. 10 and a schematic in Supplementary Fig. 11) that is consistent with the 3D morphology of 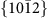 twin variants reported for Mg alloy AZ31 (ref. 19), and also would explain our observation of the large deviation between the habit plane of the DS facet and the *K*_1_ plane (Fig. 3c). In addition, as discussed by Yu *et al*.^27^, two of the three crystallographically possible 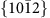 twin–twin junctions involve interactions with the DS of the twin. As a consequence, we foresee that the DS will play a role in the formation and the strength of twin–twin junctions, and on the mechanical response of Mg during cyclic deformation.

The work presented here characterizes and throws light on a novel aspect of twinning, namely, the configuration and mobility of the DS, and the role that it plays on overall twin propagations. We believe that this work will motivate further experimental, theoretical and numerical 3D characterization of twinning-associated boundaries in crystalline materials, and will lead to a better understanding of the mechanism of twinning and its contribution to deformation.

## Methods

### Sample preparation and high-resolution TEM studies

A commercially pure, fully recrystallized polycrystal Mg plate with a strong basal texture component parallel to the plate through thickness direction was strained in compression in an in-plane direction to a total strain of 1%. The electropolished samples were cut 45° to the compression direction and predominant (0001) texture fibre such that many twins would be viewed approximately down the DS. Samples were electropolished in a solution of 2% nitric acid and water at a voltage of <1 V. An FEI DB235 dual-beam focused ion beam was used to prepare cross-sectional TEM specimens from a Mg single crystal that was grown using the Bridgman method, where 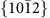 deformation twins were introduced by compression–tension cyclic loading at 1% strain amplitude. An FEI Tecnai F30 field emission transmission electron microscope with accelerating voltage of 300 kV was used for low-resolution TEM imaging. The Tecnai and an FEI Titan transmission electron microscope with an imaging aberration corrector and accelerating voltage of 300 kV were used for HRTEM imaging.

### Atomistic simulation

We examined the DS structure of the TBs by performing MD simulations with the empirical interatomic potential for Mg^36^. The MD simulation cell has the dimensions of 40, 30 and 3.2 nm with respect to the *x*, *y* and *z* directions. Periodic boundary conditions are applied along the *x* direction and *z* direction, and fixed boundary condition for the *y* direction to mimic an infinite medium. A twin domain with dimensions of 20 and 12 nm along the *x* and *y* directions was introduced by rotating the domain 180° about the normal to the twinning plane ([0,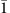,1,2/*λ*], where *λ* is 1.326 for Mg). The MD simulation was performed at a temperature of 10 K for 100 ps and followed by quenching MD until the maximum force acting on each atom is <5 pN.

## Additional information

**How to cite this article:** Liu, Y. *et al*. Characterizing the boundary lateral to the shear direction of deformation twins in magnesium. *Nat. Commun.* 7:11577 doi: 10.1038/ncomms11577 (2016).

## Supplementary Material

Supplementary Information Supplementary Figures 1-11, Supplementary Notes 1-6 and Supplementary References

Supplementary Movie 1 Molecular dynamics (MD) simulation demonstrates the pinning effect of misfit dislocations on migration of twist prismatic-prismatic (T-PP2) interfaces. Each segment of T-PP2 interfaces migrates with different velocity, resulting in irregular shape of the dark side. Misfit dislocations move associated with the propagation of T-PP2 interfaces. MD simulation was conducted at temperature of 10 K under shear strain rate of 108s-1. The position of misfit dislocations is circled as indicated in Supplementary Figure 10.
