# Supplementary material for: Characterizing the boundary lateral to the shear direction of deformation twins in magnesium
Source: Nat Commun. 2016 Jun 1;7:11577. doi: 10.1038/ncomms11577 (PMC4895437; doi:10.1038/ncomms11577)
Supplement: Supplementary Information — Supplementary Figures 1-11, Supplementary Notes 1-6 and Supplementary References [file ncomms11577-s1.pdf]

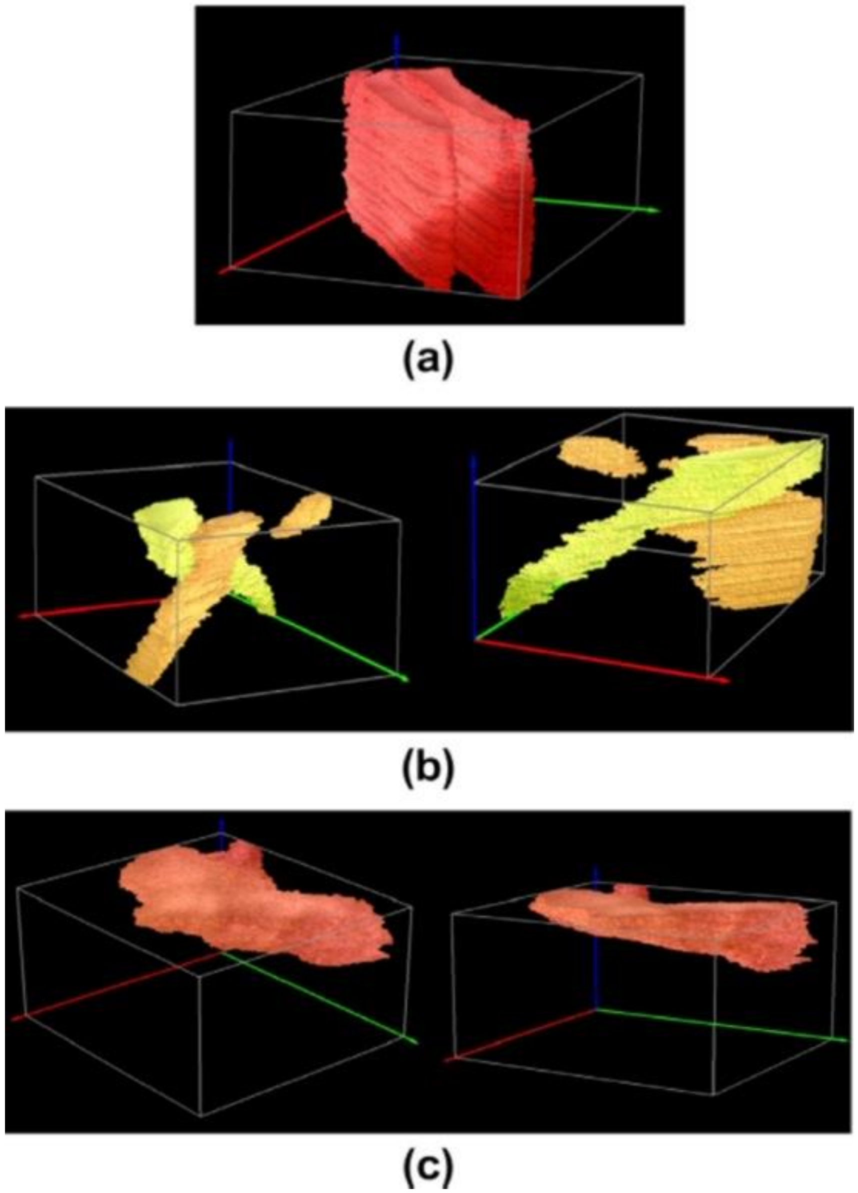

Supplementary Figure 1. **Three-dimensional morphology of twins in magnesium alloys**. The morphology of deformation twins is usually examined using two-dimensional microscopy such as optical microscope and electron backscatter diffraction (EBSD) analysis. Recently, Fernández et al reconstructed morphology of $\{10\bar{1}2\}$ deformation twins in magnesium (Mg) alloys using three-dimensional (3D) EBSD-based orientation microscopy by serial sectioning with a focused ion beam (FIB) [1]. (a) Primary twin variants with large Schmid factor, (b) secondary (yellow) and tertiary (orange) twin variants with small Schmid factor and (c) primary twin variants with small Schmid factor. The results show that twin variants with small Schmid factor may have more tortuous shapes.

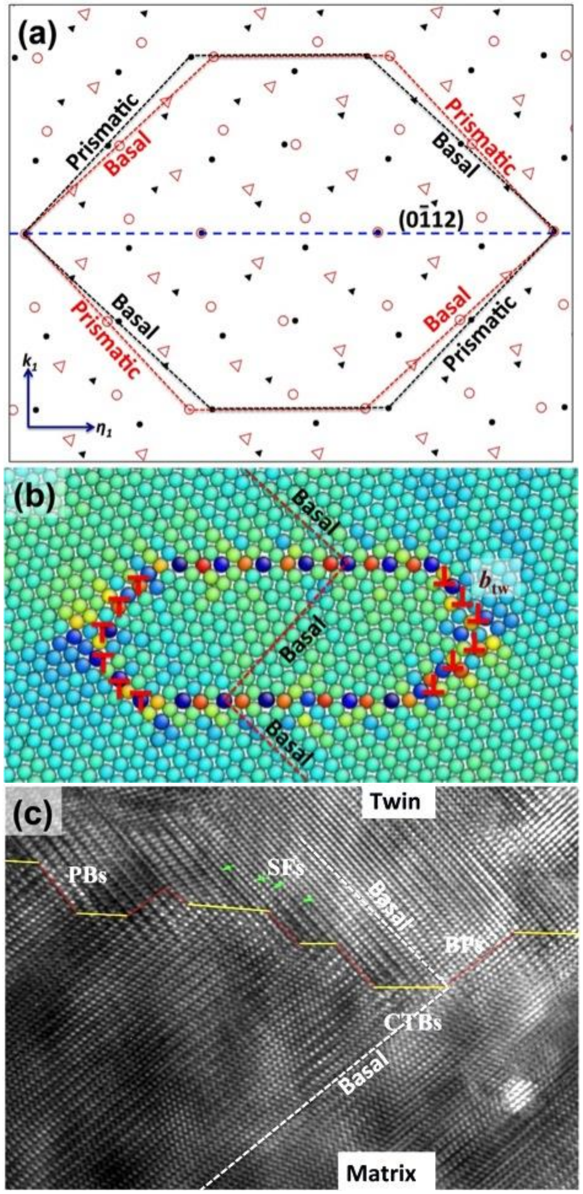

Supplementary Figure 2. **Bright side of twin boundaries**. Bright side of a twin domain shows two types of boundaries, coherent twin boundaries (CTBs) and prismatic||basal boundaries (PB/BPs). (a) The coherent dichromatic complex (CDP) of $\{10\bar{1}2\}$ twin in hexagonal close packed structure shows two geometrically preferred twin boundaries: CTBs and PB/BPs, with high atomic areal density. (b) Molecular dynamics (MD) simulations show that a twin nucleus is surrounded by CTBs and PB/BPs boundaries. The PBs/BPs are associated with the pileup of twinning dislocations [2,3]. (c) A high-resolution transmission electron microscopy (HRTEM) image shows CTBs (yellow lines) and PB/BPs (red lines) [4]. More TEM images and MD simulations can be found in supplementary references[5-7].

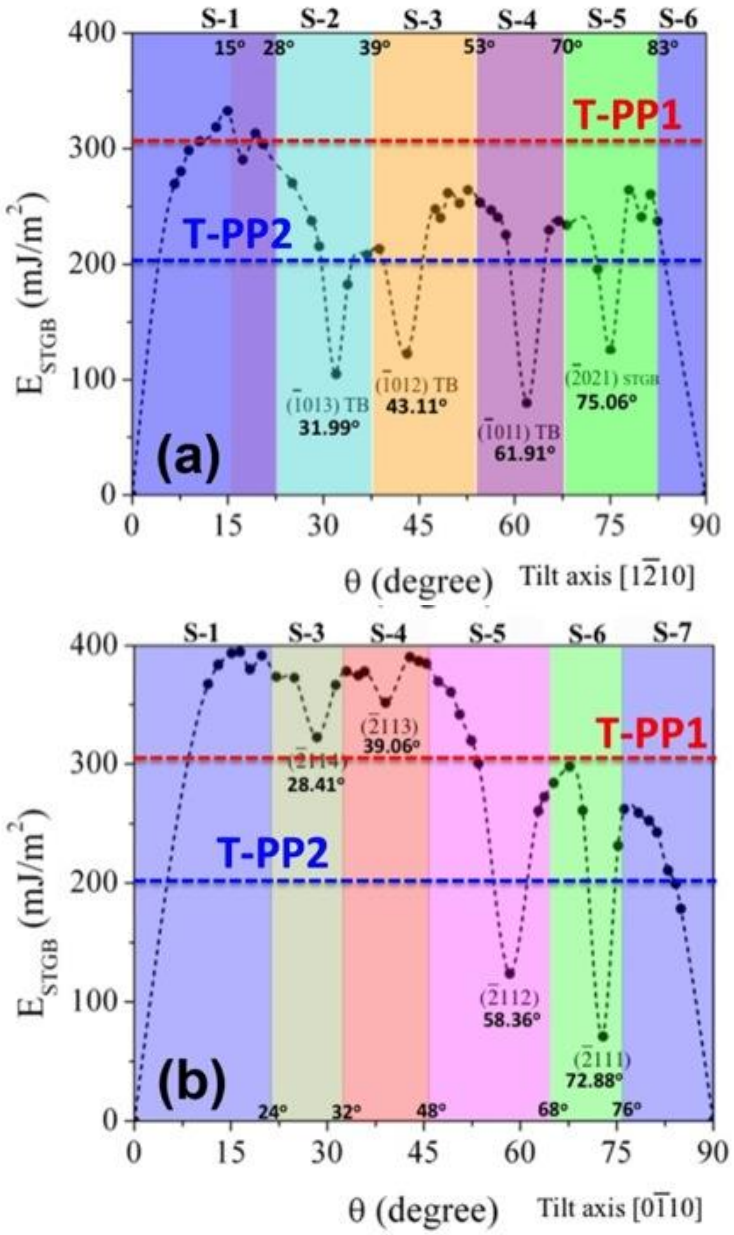

Supplementary Figure 3. **The excess potential energies of various boundaries**. Atomistic simulations with empirical potential for Mg show the excess potential energies of the twist pyramidal-pyramidal (T-PP1) and twist prismatic-prismatic (T-PP2) boundaries, 318 mJ/m^2 and 212 mJ/m^2, respectively. Compared with the excess potential energies of two groups of symmetrical tilt grain boundaries with tilt axes $< 1\bar{2}10 >$[8] and $< 0\bar{1}10 >$[9], the T-PP2 interface has low formation energy while T-PP1 interface has high formation energy.

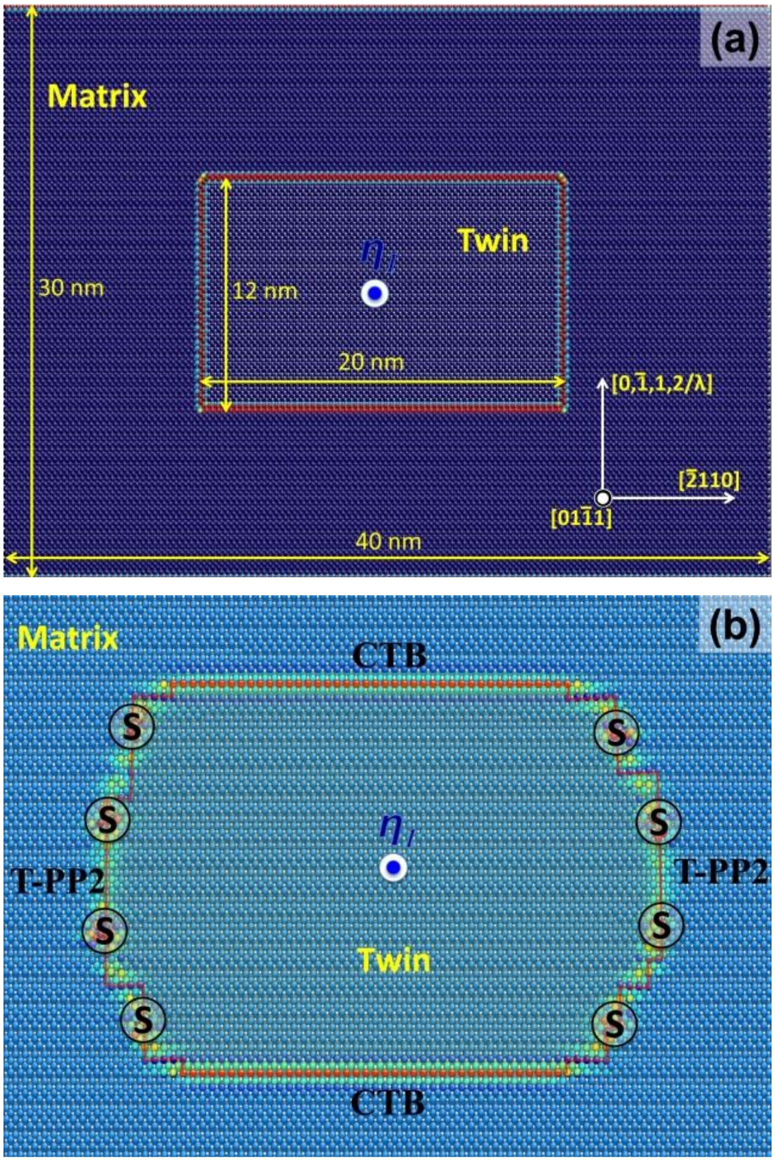

Supplementary Figure 4. **MD simulations of the dark side of a twin nucleus.** (a) Simulation cell and (b) relaxed atomic structure of a twin nucleus. The twin nucleus is surrounded by coherent twin boundaries, twist prismatic-prismatic (T-PP2) boundaries, and misfit screw dislocations. The symbol "*S*" represents misfit dislocation.

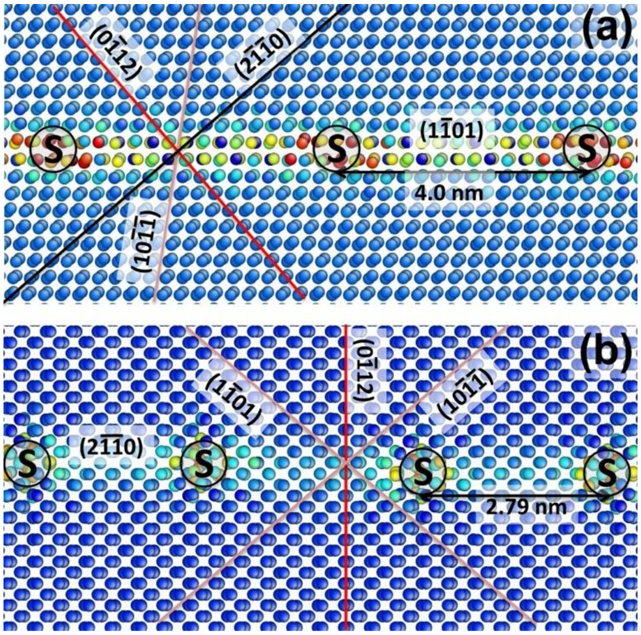

Supplementary Figure 5. **Atomic structures of T-PP1 and T-PP2 boundaries**. (a) T-PP1 and (b) T-PP2 boundaries. The symbol "S" represents misfit screw dislocation. Atoms are colored according to the excess energy. The interface region between misfit dislocations is coherent.

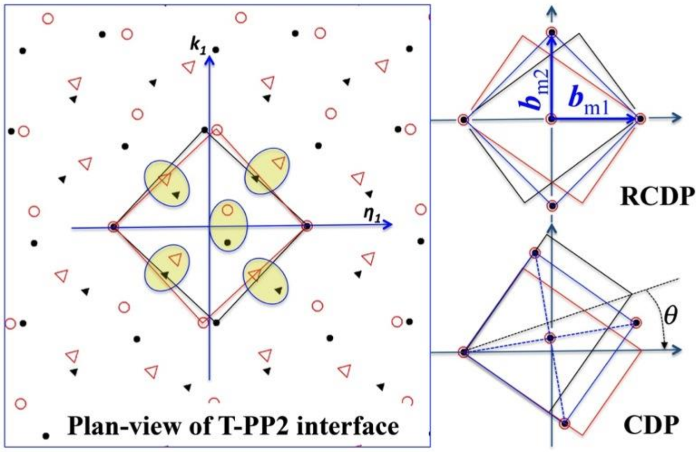

Supplementary Figure 6. **Crystallographic analysis of T-PP2 interface**. Plan-view of T-PP2 interface showing dichromatic complex of two prismatic planes from the twin domain (black solid symbols) and the matrix (red empty symbols) with respect to $\{01\bar{1}2\}$ twin orientation. The Red rectangle and the Black rectangle represent a unit hexagonal cell. The shadowed region indicates that the two atoms will occupy the same position accompanying atoms shuffle. CDP indicates the coherent dichromatic pattern of prismatic-prismatic (PP) interface associated with a twist rotation of 90°. The short side is equal to c, and the long side is equal to $\sqrt{3}a$. Corresponding to the twin orientation, PP interface experiences an additional twist rotation of θ=3.78^{o}. Thus the T-PP2 interface has the reference lattice of rotation CDP (RCDP).

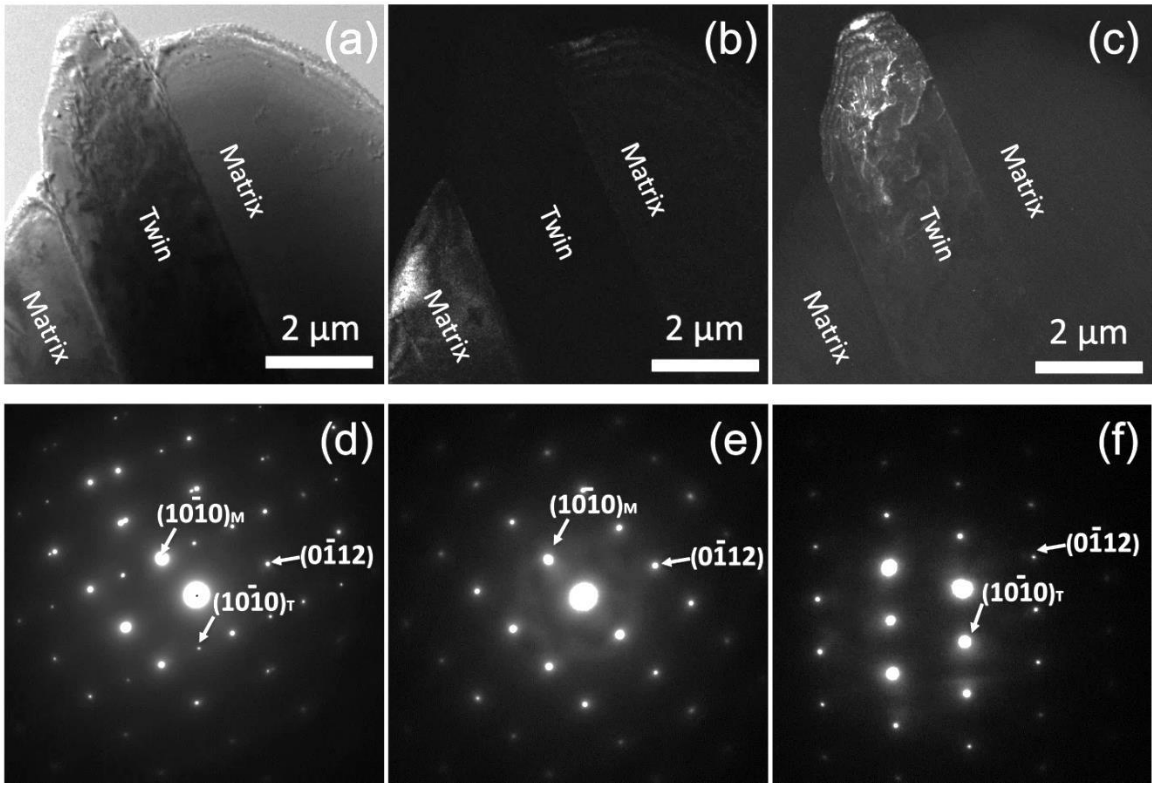

Supplementary Figure 7. **Confirm the twin relation from a non-dark side zone axis.** TEM images and corresponding selected-area diffraction (SAD) patterns observing along $[\bar{2}4\bar{2}3]$ zone axis (approximately 23° to 'dark side') confirm the presence of the $(0\bar{1}12)$ deformation twins. (a-c) Bright-field TEM in (a) and dark-field TEM of matrix domains in (b) and a twin domain in (c) revealing the twin relation. (d) SAD patterns for entire area reveal the twin relation when observing along $[\bar{2}4\bar{2}3]$. Moreover, SAD patterns of a matrix domain (e) and a twin domain (f) can be identified, respectively.

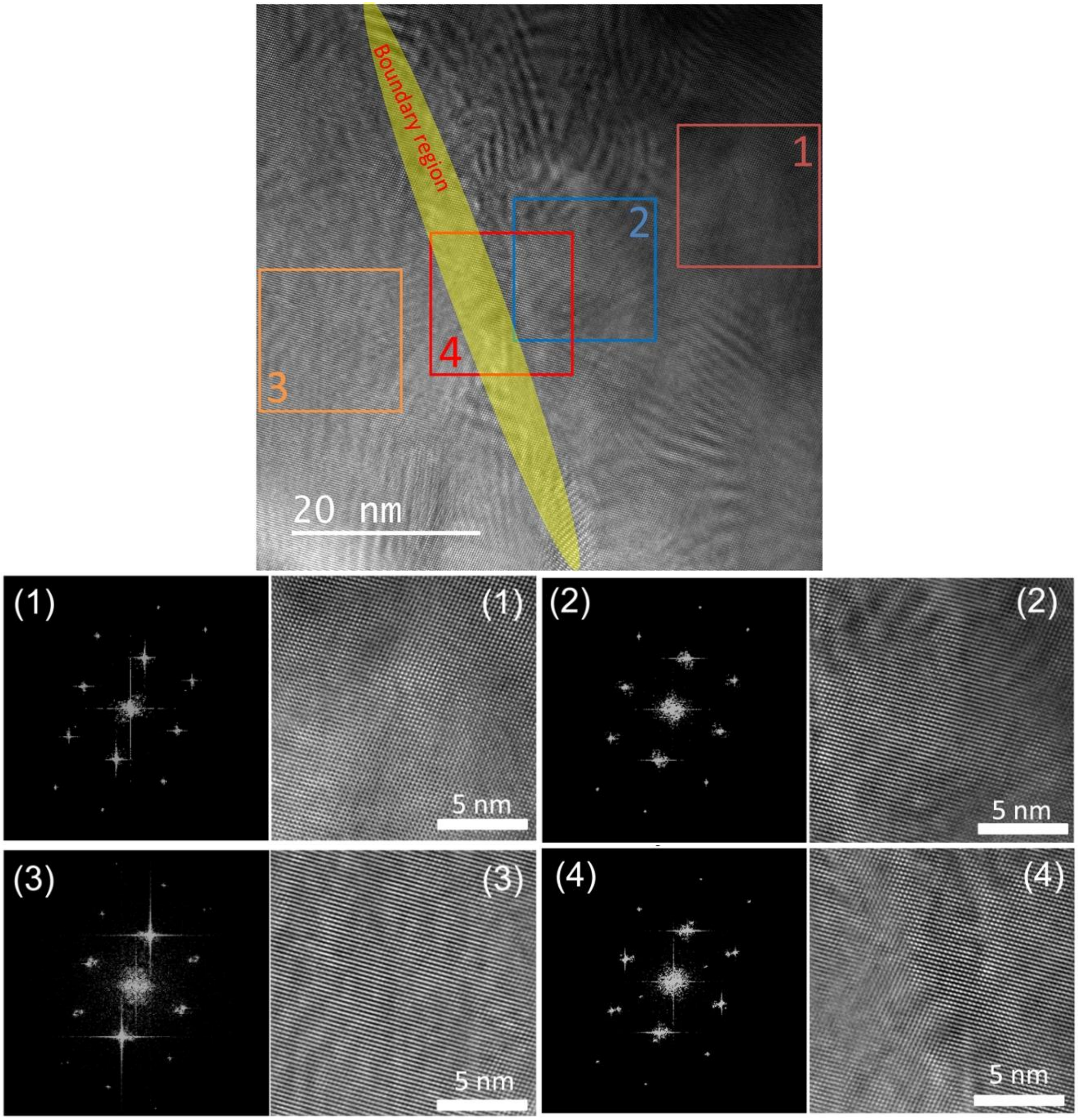

Supplementary Figure 8. **Locate the dark side of twin boundaries**. HRTEM image of Mg with matrix domain perfectly aligned, where the boundary region can only be roughly located in yellow oval area due to strain contrast and references from low-magnification images. Four different areas that are marked in the HRTEM image are analyzed: 1. Perfectly aligned matrix domain without extra diffraction information in corresponding Fast-Fourier Transform (FFT) patterns; 2. Right next to twin boundaries, the area is still perfectly aligned matrix domain without extra diffraction information in FFT patterns; 3. Slightly off-zone twin domain reveal lattice fringes and extra ($0\bar{1}12$) diffraction information in corresponding FFT patterns; 4. HRTEM on boundary region reveals extra diffraction information on ($1\bar{1}01$) plane, which provides necessary information to locate boundary serrations. HRTEM studies in Figure 3c-3e/Supplementary Figure 9 are extracted from area 4.

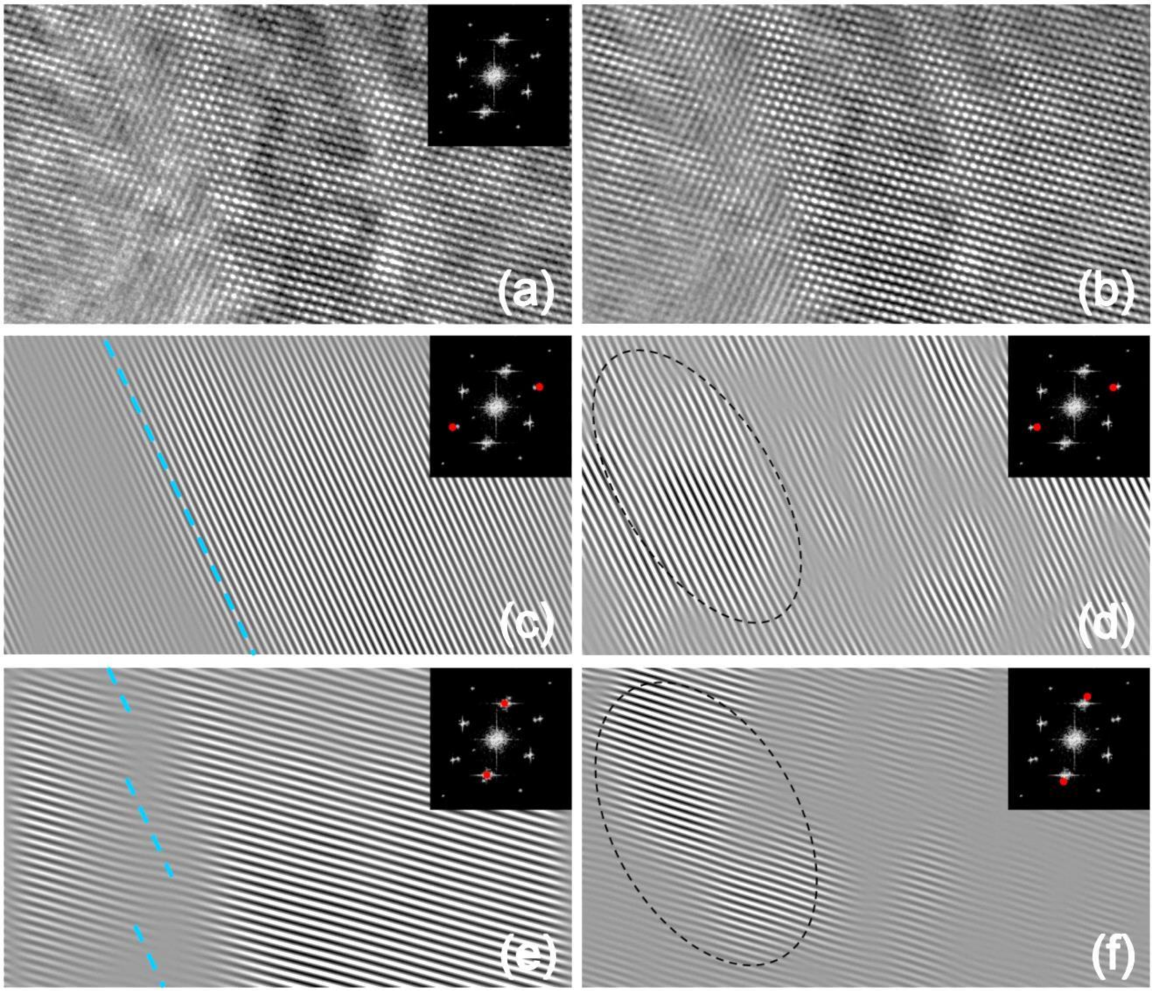

Supplementary Figure 9. **Characterize the dark side of twin boundaries**. (a) Originally captured HRTEM and corresponding FFT patterns. (b) Image processed HRTEM that used in Figure 3(c) maintained all atomic information compared in (a) with minimum artifact effects. (c-d) Inverse IFFT HRTEM images processed on $(0\bar{1}12)$ diffraction from (c) regular spots and (d) additional spots show the perfectly aligned $(0\bar{1}12)$ planes in matrix domain on the right that contributed from regular spots, while slightly off-aligned $(0\bar{1}12)$ planes in twin domain on the left as indicated by the black circle in (d) that contributed from additional spots. (e-f) IFFT HRTEM images processed on $(1\bar{1}01)$ diffraction from (e) regular spots and (d) additional spots show the discontinuity at previous marked boundary location, which is attributed to the effects from additional spots.

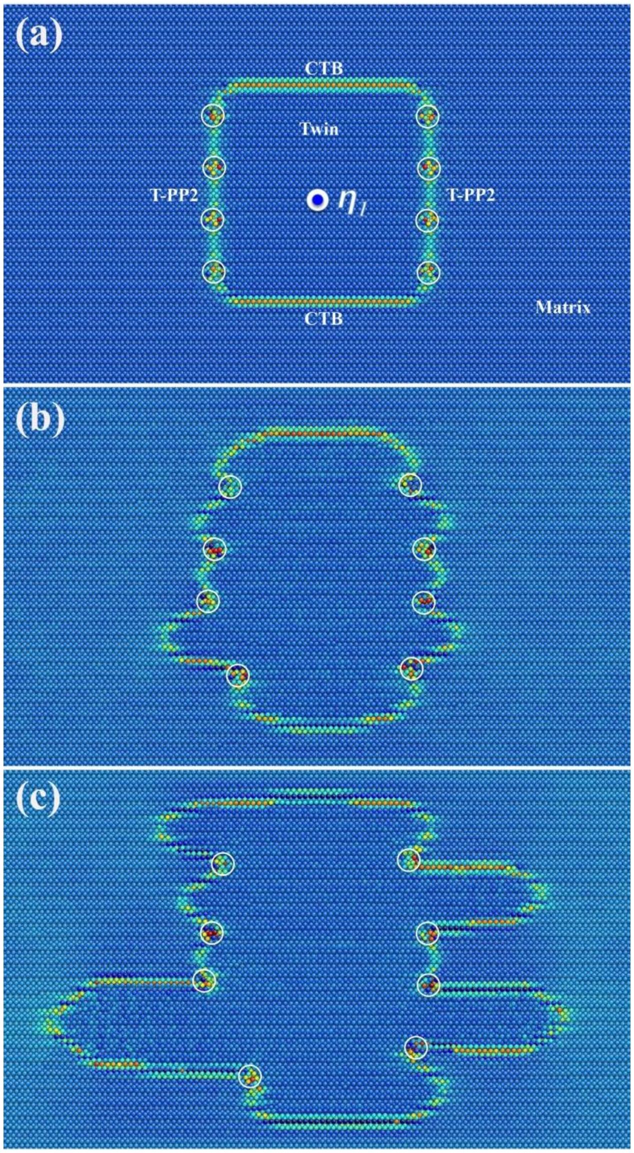

Supplementary Figure 10. **The irregular shape of T-PP2 boundary**. Molecular dynamics simulation demonstrates the pinning effect of misfit dislocations on migration of T-PP2 interfaces. Each segment of T-PP2 interfaces migrates with different velocity, resulting in irregular shape in the Dark Side. Misfit dislocations move associated with the propagation of T-PP2 interfaces. MD simulation was conducted at temperature of 10 K under shear strain rate of 10^8. The circles indicate the position of misfit dislocations. The details can be found in Movie I. The simulation cell has the dimensions of 60 nm × 23.5 nm, containing a twin with the dimensions of 12 nm ×12 nm in the horizontal and vertical directions.

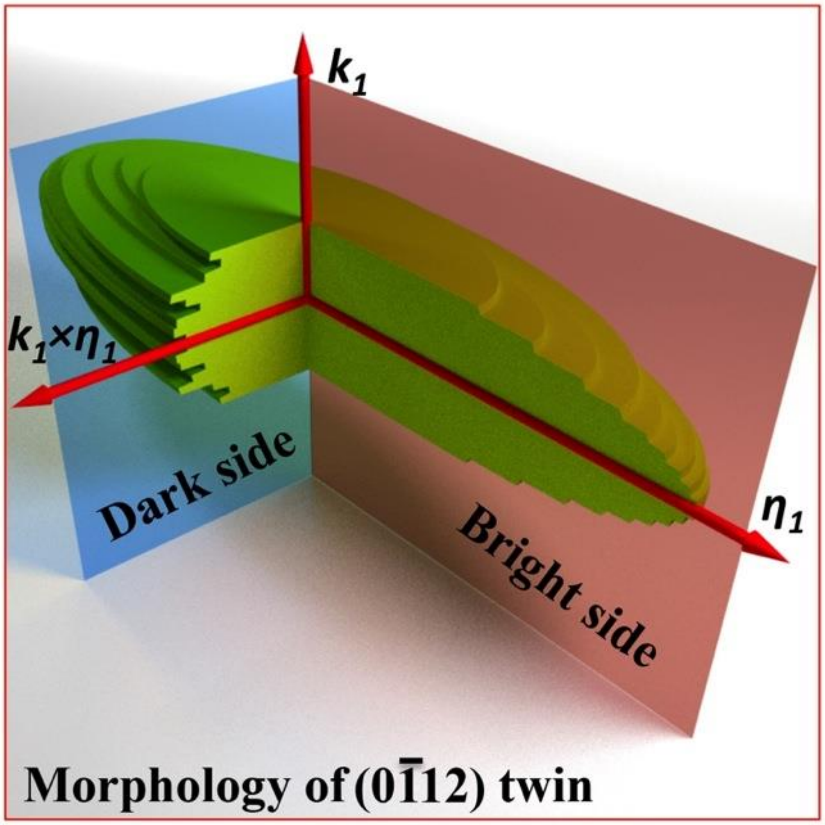

Supplementary Figure 11. **The prediction of 3D morphology of a $\{10\bar{1}2\}$ twins in Mg**. In the Bright side, twin propagates through the glide of twinning dislocations along the twinning direction (pure edge). Twin boundaries are composed of coherent twin boundary and prismatic-basal interfaces (PB/BP steps). In the Dark side, twin propagates through the migration of semi-coherent T-PP2 interface. Due to the pinning effect of misfit dislocations, each segment of T-PP2 interfaces migrates with different velocity, resulting in irregular shape.

Supplementary Note 1

MD simulations of the morphology of the dark side. We examined the Dark Side structure of the twin boundaries by performing molecular dynamics (MD) simulations with the empirical interatomic potential for Mg[10]. The MD simulation cell has the dimensions of 40 nm, 30 nm, and 3.2 nm with respect to the *x*, *y*, and *z* directions. Periodic boundary conditions are applied along the *x*-direction and z-direction, and fixed boundary condition for the *y*-direction to mimic an infinite medium. A twin domain with dimensions of 20 nm and 12 nm along the *x* and *y* directions was introduced by rotating the domain 180° about the normal to the twinning plane ($[0,\bar{1},1,2/\lambda]$, where λ is 1.326 for Mg) (Supplementary Figure 4a). The MD simulation was performed at a temperature of 10 K for 100 pico-seconds and followed by quenching molecular dynamics until the maximum force acting on each atom is less than 5 pN. The MD simulation reveals two types of twin boundaries: coherent twin boundary (CTB) and the T-PP2 semi-coherent boundaries in which misfit dislocations are present (Supplementary Figure 4b). The details of semi-coherent T-PP2 boundary and misfit dislocations will be addressed in what follows.

Supplementary Note 2

MD simulations of T-PP1 and T-PP2 boundaries. We studied atomic structures of twist pyramidal-pyramidal boundaries (T-PP1) and twist prismatic-prismatic (T-PP2) boundaries by performing MD simulations with an empirical interatomic potential for Mg[10]. For the T-PP1 interface, the top crystal coordinates are (λ is 1.326 for Mg): the *x*-axis lies along $[2+\lambda,\lambda-1,-1-2\lambda,\bar{3}]$, the *y*-axis along $[1,\bar{1},0,1/\lambda]$ and the *z*-axis along <$01\bar{1}1$>, while in the bottom crystal the *x*-axis lies along $[-2-\lambda,1-\lambda,1+2\lambda,3]$, the *y*-

axis along $[\bar{1},1,0,\bar{1}/\lambda]$ and the z-axis along $<01\bar{1}1>$. For the T-PP2 interface, the top crystal coordinates are: the x-axis lies along $[0,\bar{1},1,2/\lambda]$, the y-axis along $[2\bar{1}\bar{1}0]$, and the z-axis along $<01\bar{1}1>$, while in the bottom crystal the x-axis lies along $[0,1,\bar{1},\bar{2}/\lambda]$, the y-axis along $[\bar{2}110]$, and the z-axis along $<01\bar{1}1>$. The corresponding MD simulation cell contains a single boundary on the x-z plane. The simulation model contains two parts — a moveable region inside the simulation cell and a semi-rigid region that surrounds the moveable region. The semi-rigid region acts as a flexible boundary to mimic the bulk response during relaxation[11]. Periodic boundary conditions are applied in both the x and z directions. The x dimension varies depending on the boundary type such that periodic boundary conditions are satisfied. The height of the two crystals along y is 10.0 nm and the thickness of semi-rigid regions alomg y is 1.2 nm, which is two times the cutoff of the potential used. The dimension in the z direction is ~3.2 nm containing 10 periodic units.

The bicrystal simulation model is relaxed at 0 K by quenching molecular dynamics (MD) using an embedded atom method (EAM) potential for Mg. During relaxation, the two crystals are allowed to translate in the three orthogonal x-y-z directions, but restricted from rotating about the boundary normal. Relaxation ends when the maximum force acting on any atom in the system does not exceed 5 pN. Atomistic simulations reveal the energies and atomic structures of the three interfaces. CTB has the lowest formation energy of 125 mJ/m^2, T-PP2 has a formation energy of 212 mJ/m^2, which is lower than most tilt GBs, and T-PP1 has the highest formation energy of 318 mJ/m^2, which is higher than most tilt GBs (also see Supplementary Figure 3). Supplementary Figure 5 shows the atomic structures of T-PP1 and T-PP2 interfaces, indicating formation of semi-coherent interface and misfit dislocations.

Supplementary Note 3

Formation mechanisms of semi-coherent T-PP2 boundary. The concept of misfit dislocations relieving coherency strain is well known in dislocation theory[12]. Recently, J. P. Hirth et al clarified how to define misfit dislocations[13]. In general, the net Burgers vector **B** in an interface can be described by the Frank-Bilby equation[14,15], and the discrete misfit dislocations can be well defined by the atomistically-informed Frank-Bilby method[16].

Atomistic simulations reveal that the T-PP2 prism-prism interface consists of coherent interface regions separated by misfit dislocations, i.e., a semi-coherent interface. This can be explained based on formation and reaction of interface dislocations. The detailed theoretical description can be found in Ref.13. Atomistic simulations using density function theory and molecular statics method[17-19] indicated a low energy coherent Prismatic-Prismatic (PP) interface where the prismatic plane of the twin is twisted 90° with respect to the prismatic plane of the matrix. The T-PP2 interface (with a 86.22^o twist associated with the twin orientation) relaxes into the coherent PP interface (90° twist) plus misfit dislocations that accommodate the additional twist rotation 3.78^o. Corresponding to the dislocation conservation at the interface, discrete misfit dislocations that formed on the interface are balanced by uniform distribution of dislocations that make the original interface coherent.

The mean separation between discrete misfit dislocations in the interface is given by the Frank formula[13], $d = 2b_{misift} \tan(\theta/2)$. Here, θ is the twist angle and equals 3.78^o, and b_{misift} is the discrete misfit dislocation Burgers vector and the magnitude is equal to

$(c+\sqrt{3}a)/2\sqrt{2}$. The Burgers vector of the discrete misfit dislocations can be determined based on a reference lattice, i.e. coherent dichromatic pattern (CDP).

A reference CDP for a misfit dislocation is presented in Supplementary Figure 6 where the coherency strain is partitioned between the crystals. The T-PP2 interface has a rotation 3.78^{o} with respect to the reference lattice CDP, thus a rotation CDP (RCDP) is defined in Supplementary Figure 6[13]. There are two possible Burgers vectors, b_{m1} and b_{m2}. For Mg, the magnitude of the two Burgers vectors is equal to 0.378 nm. According to the Frank formula, we obtain the mean separation between discrete misfit dislocations, d=2.88 nm, which is consistent with our MD result, d=2.79 nm.

Supplementary Note 4

Determine dark side's boundary regions at low magnification in TEM. The dark side of a Compound $(01\bar{1}2)$ twin in Mg is examined along the η_1 direction ($[01\bar{1}1]$ zone axis) and $[\bar{2}4\bar{2}3]$ zone axis. As suggested by diffraction simulation, the SAD of twin and matrix domains are identical when observing along $[01\bar{1}1]$ ('dark side'). However, low-magnification bright-field TEM image in Figure 3a-3b showing distinct twin and matrix domains when observing along $[01\bar{1}1]$ ('dark side'). This due to a deviation from the perfect twin relation, when the parent is perfectly aligned with the zone axis, the twin domain is tilted slightly off of the zone axis (~1°). This misorientation helps the identification of twin domains from matrix domain due to the thickness fringes differences.

We then examine the same area along $[\bar{2}4\bar{2}3]$ zone axis to confirm the twin relation, since the SAD patterns of twin and matrix domain are not identical. As shown in Supplementary Figure 7 both bright-field and dark-field TEM images and corresponding

SAD patterns confirms the twin relation when observing along $[\bar{2}4\bar{2}3]$ zone axis. Therefore, we have successfully confirm the presence of the $\{10\bar{1}2\}$ deformation twins. Moreover, the local misorientation between twin and matrix enables the finding of the 'dark side' boundary at low-magnification.

Supplementary Note 5

HRTEM and FFT analysis. Once the boundary region is roughly located, we characterize a small region marked by the red square with HRTEM. As shown in Supplementary Figure 8, HRTEM image of Mg with matrix domain perfectly aligned, where the boundary region can only be roughly located within the yellow oval area due to the strain contrast and references from low-magnification images. Four different areas that are marked in the HRTEM image are analyzed: 1. Perfect aligned matrix domain without extra diffraction information in corresponding Fast-Fourier Transform (FFT) patterns; 2. Right next to twin boundaries, the area is still in well-aligned matrix domain without extra diffraction information in FFT patterns; 3. Slightly off-zone twin domain reveal lattice fringes and extra $(0\bar{1}12)$ diffraction information in corresponding FFT patterns; 4. HRTEM on boundary region reveals extra diffraction information on $(1\bar{1}01)$ plane, which provides necessary information to locate boundary serrations. HRTEM studies in Supplementary Figure 9/Figure 3c-3e are focused on area 4.

Supplementary Note 6

Inverse FFT (IFFT) analysis. The determination of the boundary serrations is based on Inverse FFT (IFFT) analysis on the misorientation induced extra diffraction information of the $(0\bar{1}12)$ and $(1\bar{1}01)$ planes. An IFFT image that is extracted from $(0\bar{1}12)$ diffraction spots, as shown in Figure 3d, shows well-aligned $(0\bar{1}12)$ planes in matrix domain on the

right side and slightly off-aligned $(0\bar{1}12)$ planes in twin domain on the left side. The blue dashed-line on a $(0\bar{1}12)$ plane roughly indicates the possible boundary location. Then, an IFFT image that is extracted from $(1\bar{1}01)$ diffraction spots in Figure 3e shows the discontinuity at the previous marked boundary location, which can be differentiated into several segments of CTBs. At the end, the local displacement of atoms that created by screw dislocation cores can be identified as shown in Figure 3c, suggesting the serration of the twin boundaries observing along the 'dark side'.

Detail analysis can be found in Supplementary Figure 9. Compared to originally captured HRTEM in Supplementary Figure 9a, image processed HRTEM (Supplementary Figure 9b) that used in Figure 3c maintained all atomic information with minimum artifact effects. IFFT HRTEM images processed on $(0\bar{1}12)$ diffraction spots from Supplementary Figure 9c regular spots and Supplementary Figure 9d additional spots show the well-aligned $(0\bar{1}12)$ planes in matrix domain on the right that contributed from regular spots, while slightly off-aligned $(0\bar{1}12)$ planes in twin domain on the left as indicated by the black dash-line circle. The possible boundary location can be identified by the blue dash-line. Then, IFFT HRTEM images processed on $(1\bar{1}01)$ diffraction from Supplementary Figure 9e regular spots and Supplementary Figure 9d additional spots show the discontinuity at previous marked boundary location, which is attributed to the effects from additional spots. Therefore, the 'dark side' boundaries with serrations can be successfully identified.

Supplementary References

1. Fernández, A., Jérusalem, A., Gutiérrez-Urrutia, I. & Pérez-Prado M. T. Three-dimensional investigation of grain boundary–twin interactions in a Mg AZ31 alloy by electron backscatter diffraction and continuum modeling. *Acta Mater* **61**, 7679-7692 (2013).
2. Wang, J. *et al.* Nucleation of a twin in hexagonal close-packed crystals. *Scripta Mater* **61**, 903-906 (2009).
3. Wang, J., Hirth, J. P. & Tomé C. N. Twinning nucleation mechanisms in hexagonal-close-packed crystals. *Acta Mater* **57**, 5521-5530 (2009).
4. Tu, J. *et al.*, Structural characterization of {10-12} twin boundaries in cobalt. *Appl Phys Lett* **103**, 051903 (2013).
5. Barrett C. D. & El Kadiri H. Impact of deformation faceting on {10-12}, {10-11} and {10-13} embryonic twin nucleation in hexagonal close-packed metals. *Acta Mater* 2014, **70**: 137-161.
6. Ostapovets A. & Serra A. Characterization of the matrix–twin interface of a (10-12) twin during growth. *Phil Mag* 2014, **94**: 2827-2839.
7. Xu B., Capolungo L. & Rodney D. On the importance of prismatic/basal interfaces in the growth of twins in hexagonal close packed crystals. *Scrip Mater* 2013, **68**: 901-904.
8. Wang, J. & Beyerlein, I. J. Atomic structures of symmetric tilt grain boundaries in hexagonal close packed (hcp) crystals. *Modeling and Simulation in Materials Science and Engineering* **20**, 024002 (2012).
9. Wang, J. & Beyerlein, I. J. Atomic Structures of $[0\bar{1}10]$ Symmetric Tilt Grain Boundaries in Hexagonal Close-Packed (hcp) Crystals. *Metallurgical and Materials Transactions A* **43**, 3556-3569 (2012).
10. Liu, X. Y., Adams, J. B., Ercolessi, F. & Moriarty, J. A. EAM potential for magnesium from quantum mechanical forces. *Modeling and Simulation in Materials Science and Engineering* **4**, 293 (1996).
11. Wang, J., Hoagland, R. G., Hirth, J. P. & Misra, A. Atomistic simulations of the shear strength and sliding mechanisms of copper-niobium interfaces. *Acta Mater* **56**, 3109-3119 (2008).
12. Sutton, A. P. & Balluffi R. W. *Interfaces in Crystalline Materials* (Oxford University Press, Oxford, 1995).
13. Hirth, J. P., Pond, R. C., Hoagland, R. G., Liu, X. Y. & Wang, J. Interface defects, reference spces and the Frank-Bilby equation. *Progress in Materials Science* **58**, 749-823 (2013).
14. Frank, F. C. Report of the Symposium on the Plastic Deformation of Crystalline Solids (Carnegie Institute of Technology, Pittsburgh, PA, 1950), p. 150.
15. Bilby, B. A. Report of the Conference on Defects in Crystalline Solids (Physical Soc, London; 1955), p. 124.
16. Wang, J., Zhang, R. F., Zhou, C. Z., Beyerlein, I. J. & Misra A. Characterizing interface dislocations by atomically informed Frank-Bilby theory. *Journal of Materials Research* **28**, 1646-1657 (2013).

17. Wang, J., Yadav, S. K., Hirth, J. P., Tomé, C. N. & Beyerlein, I. J. Pure-shuffle nucleation of deformation twins in hexagonal-close-packed metals. *Materials Research Letters* **1**, 126-132 (2013).
18. Kumar, A., Wang, J. & Tomé, C. N., First-principles study of energy and atomic solubility of twinning-associated boundaries in hexagonal metals, *Acta Mater* **85**, 144-154 (2015).
19. Yu, Q., Wang, J., Jiang, Y., McCabe, R. J. & Tomé, C. N. Co-zone $\{\bar{1}012\}$ Twin Interaction in Magnesium Single Crystal. *Materials Research Letters* **2**, 82-88 (2014).
